# Supplementary material for: Allosteric disulfide control of ligand binding and endocytosis of KIR2DL4, the natural killer cell receptor for HLA-G
Source: eLife. 2026 Oct 13;15:RP111018. doi: 10.7554/eLife.111018 (PMC13661046; doi:10.7554/eLife.111018)
Supplement: Supplementary file 2 [file elife-111018-fig3-data1.docx]

2DL4 peptides analyzed to determine disulfide bond redox state. Peptides were detected by Byonic analysis software, confirmed by MS/MS and have errors <6 ppm. Only peptides with peak areas >10 million for a given Cys were included in the analysis.

| **Cys** | **Peptide sequence*** | **Peptide score^#^** | **Peptide error (ppm)** |
| --- | --- | --- | --- |
| 10 | **C**SAWPSAVVPQGGHVTL  **C**SAWPSAVVPQGGH  **C**SAW | 549.9  403.3  351.1 | 0.16  0.8  0.8 |
| 28 | R**C**HY | 255.5 | 0.3 |
| 74 | R**C**RGF | 245.7 | 0.72 |
| 123 | S**C**SSQSSF  S**C**SSQSSFDIY | 383.9  387.2 | 0.97  0.32 |
| 172 | R**C**FGSF | 367.9 | 0.91 |

*Cys was labelled with ^12^C-iodoacetanilide or ^13^C-iodoacetanilide and is in bold.

^#^Peptide score calculated by Byonic analysis software is a primary indicator of peptide-spectrum matches.
