# Supplementary material for: Allosteric disulfide control of ligand binding and endocytosis of KIR2DL4, the natural killer cell receptor for HLA-G
Source: eLife. 2026 Oct 13;15:RP111018. doi: 10.7554/eLife.111018 (PMC13661046; doi:10.7554/eLife.111018)
Supplement: Supplementary file 3 [file elife-111018-fig6-data1.docx]

| **Epitope** | **HLA** | **KIRs** | **K_D_ (**μM**)** |
| --- | --- | --- | --- |
| Pp65-2-VLPHETRLL | HLA-G wt | KIR2DL4*001 | 19.4 μM ± 4.00 |
| Pp65-2-VLPHETRLL | HLA-G wt | KIR2DL4*001C10L | 1.59 ± 0.08 |
| Pp65-2-VLPHETRLL | HLA-GC42S | KIR2DL4*001C10L | 1.65 ± 0.07 |
| Pp65-2-VLPHETRLL | HLA-G wt | KIR3DL1*001 | >300 |
| Pp65-2-VLPHETRLL | HLA-GC42S | KIR3DL1*001 | >300 |
| H2A-RIIPRHLQL | HLA-G wt | KIR2DL4*001 | N.B. |
| Pp65-6-VFPTKDVAL | HLA-G wt | KIR2DL4*001 | N.B. |
| H2A-RIIPRHLQL | HLA-G wt | KIR2DL4*001C10L | N.B. |
| Pp65-6-VFPTKDVAL | HLA-G wt | KIR2DL4*001C10L | N.B. |
| H2A-RIIPRHLQL | HLA-GC42S | KIR2DL4*001C10L | N.B. |
| Pp65-6-VFPTKDVAL | HLA-GC42S | KIR2DL4*001C10L | N.B. |
