# Supplementary material for: Allosteric disulfide control of ligand binding and endocytosis of KIR2DL4, the natural killer cell receptor for HLA-G
Source: eLife. 2026 Oct 13;15:RP111018. doi: 10.7554/eLife.111018 (PMC13661046; doi:10.7554/eLife.111018)
Supplement: Peer review history

# Editor report: eLife Assessment

**DOI:** 10.7554/eLife.111018.3.sa0

**Authors:** Shiny Nair

**Affiliations:** Yale University United States

**Keywords:** Important, Solid

This **important** study provides new insights into how extracellular-domain disulfide configurations regulate ligand binding and endocytosis of the natural killer cell receptor KIR2DL4, identifying a conformational switch that provides a mechanistic framework for receptor trafficking. The evidence supporting the main conclusions is **solid**, with complementary biochemical, structural, and functional approaches providing strong support for alternative disulfide configurations and their role in regulating receptor behavior. The work will be of interest to researchers studying natural killer cell biology, immune receptor signaling, and protein disulfide regulation.

# Referee report: Reviewer #1 (Public review)

**DOI:** 10.7554/eLife.111018.3.sa1

Summary:

This paper asks how the NK cell receptor KIR2DL4 binds HLA-G and undergoes endocytosis. The authors propose that an allosteric disulfide-bond switch controls whether the receptor is in a ligand-binding or non-binding state, and they support this model using mutagenesis, imaging, mass spectrometry, and structural prediction.

Strengths:

A major strength is the use of diverse, complementary approaches to validate the central claim. The authors combined unbiased random mutagenesis to identify key residues, confocal microscopy to track cellular localization , and mass spectrometry to quantify the redox states of specific disulfide bonds. These methods consistently support a single model: an allosteric disulfide switch. The transition between a Cys10-Cys28 bond and a Cys28-Cys74 bond serves as a functional switch that controls whether the receptor resides at the plasma membrane to bind ligand or remains inactive in endosomes.

Comments on revised version.

The revision substantively addresses the core weaknesses I raised, particularly on direct binding evidence, which was the most important gap. The remaining points (oligomerization confound in the SPR comparison, C10L not independently validated by imaging, and the 293T-only functional readout) are real but secondary so I'd suggest they be addressed with a sentence or two of acknowledgment in the Discussion rather than additional experiments.

# Referee report: Reviewer #2 (Public review)

**DOI:** 10.7554/eLife.111018.3.sa2

Summary:

Rajagopalan et al. shows how extracellular domain features regulate KIR2DL4 internalization. The trafficking phenotypes of cysteine mutants are logically organized and well summarized in Table. The disulfide mapping and differential alkylation strategy is appropriate and provides strong support for alternative disulfide configurations in D0. The higher accessibility or more selective reduction of Cys10-Cys28 as compared to Cys28-Cys74 by PDI is a key mechanistic anchor.

Strengths:

The identification of a conformational switch in KIR2DL4 is conceptually novel. Experimental elegance, detailed and well written.

# Author comment: Author response

**DOI:** 10.7554/eLife.111018.3.sa3

**Authors:** Sumati Rajagopalan, Joyce Chiu, Priyanka Chaurasia, Jinghua Lu, George M Mastorakos, Saurav Majumder, Kristof T Nolan, Jan Petersen, Erin J Adams, Peter D Sun, Jamie Rossjohn, Philip J Hogg, Eric O Long

**Affiliations:** National Institutes of Health Rockville United States; UNSW Sydney Sydney Australia; Monash University Clayton Australia; National Institutes of Health Rockville, Maryland United States; Scienza Health Newport Beach, California United States; NIH Bethesda United States; AstraZeneca Gaithersburg, Maryland United States; Monash University Clayton Australia; University of Chicago Chicago United States; National Institute of Allergy and Infectious Diseases Rockville United States; Monash University Clayton Australia; University of Sydney Sydney Australia; National Institute of Allergy and Infectious Diseases Rockville United States

The following is the authors’ response to the original reviews.

**Public Reviews:**

**Reviewer #1 (Public review):**

Summary:

This paper asks how the NK cell receptor KIR2DL4 binds HLA-G and undergoes endocytosis. The authors propose that an allosteric disulfide-bond switch controls whether the receptor is in a ligand-binding or non-binding state, and they support this model using mutagenesis, imaging, mass spectrometry, and structural prediction.

Strengths:

A major strength is the use of diverse, complementary approaches to validate the central claim. The authors combined unbiased random mutagenesis to identify key residues, confocal microscopy to track cellular localization, and mass spectrometry to quantify the redox states of specific disulfide bonds. These methods consistently support a single model: an allosteric disulfide switch. The transition between a Cys10-Cys28 bond and a Cys28-Cys74 bond serves as a functional switch that controls whether the receptor resides at the plasma membrane to bind ligand or remains inactive in endosomes.

Weaknesses:

(1) The core model is interesting, but some of the strongest mechanistic claims still rely heavily on structure prediction rather than direct structural evidence, especially the proposed HLA-G contact surface in Figure 6 (now in Figure 7).

The crystal structure of KIR2DL4 has a D0 domain in the C10-C28 disulfide configuration [1]. The AlphaFold prediction is different, having a C28-C74 disulfide bond in the D0 domain. It is understood that any prediction could be wrong. Nevertheless, the AlphaFold structure did point to the possibility that KIR2DL4 exists in two different disulfide-bonded forms. We went on to demonstrate experimentally that these two forms coexist in human cells. This conclusion is independent of the structure predicted by AlphaFold.

The second difference predicted by AlphaFold is an allosteric change in a loop distant from the disulfide bond, suggesting the possibility that it could control binding of HLA-G. Again, this prediction could be wrong. Nevertheless, considering that the KIR2DL4 used to obtain a crystal structure was in a C10-C28 bond configuration and did not bind HLA-G [1], we wondered if HLA-G would bind to KIR2DL4 in a C28-C74 configuration.

New experiments included in the revision have shown that a purified Cys10Leu KIR2DL4 mutant binds HLA-G (new Figure 6). Solving the structure of a KIR2DL4–HLA-G complex would be ideal, but this has not been possible thus far. The difficulty in crystallizing KIR2DL4 may be due, in part, to its propensity to form oligomers [1], as shown in the new Figure S6.

The addition of both direct binding of HLA-G to KIR2DL4 and functional data showing that KIR2DL4 induces an ISG response when in the C28-C74 but not in the C10-C28 configuration strengthens the conclusion that disulfide switching controls ligand binding and downstream signaling relevant to NK cell interactions with HLA-G in early pregnancy.

(2) The paper supports an effect of the disulfide state on trafficking and uptake, but the case for direct KIR2DL4-HLA-G binding still feels somewhat indirect. The manuscript itself notes that direct binding had not been previously shown, and the current explanation partly depends on inference about which disulfide state is present.

Direct binding and affinity measurements of HLA-G bound to the Cys10Leu KIR2DL4 mutant (in a C28-C74 disulfide form) are in the new Figure 6. This crucial result is also consistent with functional data. New experiments (new Figure 5D) have shown that the ability of HLA-G to stimulate a transcriptional interferon-stimulated gene (ISG) response occurred with the C28-C74 form, but not the C10-C28 form of KIR2DL4.

Surface plasmon resonance data showed for the first time direct binding between the C28-C74 form of KIR2DL4 and soluble HLA-G, with a K_D_ of 1.6 mM (new Figure 6). Binding to WT KIR2DL4, which is in both configurations, C10-C28 and C28-C74, was also detected but with a lower affinity (K_D_ = 19.4 mM). The purified WT KIR2DL4 formed oligomers (new Figure S6). In addition, binding of HLA-G to KIR2DL4 depended on the sequence of the peptide presented by HLA-G, as only one out of three peptides tested was compatible with KIR2DL4 binding.

This new data was obtained in the laboratory of Jamie Rossjohn at Monash University, Victoria, Australia. He, along with Jan Peterson and Priyanka Chaurasia are new co-author on our revised manuscript.

(3) Most of the main experiments are done in transfected 293T cells, so it is still not fully clear how strongly this mechanism carries over to the more relevant NK-cell setting discussed in the paper.

Primary resting NK cells are not amenable to transfection. Despite this technical hurdle, we have included two key findings with primary NK cells in the revision.

(1) As in the 293T transfected cell system, we have shown that inhibition of PDI caused reduced uptake of HLA-G in primary resting NK cells (New Figure 4E, F, G). This is consistent with uptake of HLA-G by the C28-C74 form of KIR2DL4 and with a switch from C10-C28 to C28-C74 catalyzed by PDI.

(2) We have shown that cell-surface C28-C74 KIR2DL4 on primary NK cells, as detected by mAb 2388, decreased upon inhibition of PDI, again consistent with the role of PDI in maintaining a pool of C28-C74-bonded KIR2DL4 at the cell surface (New Figure 5E, F). As shown in the original Figure 3, PDI could reduce the C10-C28 bond in purified WT KIR2DL4 in vitro.

(4) The cellular evidence for the PDI story is not specific, since it depends a lot on inhibitor and blocking experiments that could affect the broader extracellular redox environment.

Using inhibitors that target PDIA1 selectively, namely Rutin (PDI-specific up to 30 microM), and a PDI-specific monoclonal antibody, we found that HLA-G uptake by primary NK cells was inhibited (Figure 4C, D). We admit that pCMPS and thiol blockade by DTNB (Figure 4A, B) affect the extracellular redox environment. Data that were obtained without PDI inhibitors include the reduction of the C10-C28 bond by PDI in WT KIR2DL4 in vitro (Figure 3F), direct binding of HLA-G to KIR2DL4 in a C28-C74 disulfide conformation (Figure 6), and a functional transcriptional response to HLA-G by C28-C74 KIR2DL4 and not with the C10-C28 KIR2DL4.

**Reviewer #2 (Public review):**

Summary:

Rajagopalan et al show how extracellular domain features regulate KIR2DL4 internalization. The trafficking phenotypes of cysteine mutants are logically organized, and well-summarized in a Table. The disulfide mapping and differential alkylation strategy are appropriate and provide strong support for alternative disulfide configurations in D0. The higher accessibility or more selective reduction of Cys10-Cys28 as compared to Cys28-Cys74 by PDI is a key mechanistic anchor.

Strengths:

The identification of a conformational switch in KIR2DL4 is conceptually novel. Experimental elegance, detailed and well-written.

Weaknesses:

Most of the mechanistic work was shown in HEK293. The authors should exhibit relevance using primary NK cells (using primary NK)

As primary NK cells are not amenable to transfection, it is difficult to dissect the role of each disulfide form of the receptor KIR2DL4.

Instead, we have now included PDI inhibition experiments using primary NK cells and shown that PDI inhibition reduces HLA-G uptake by primary NK cells (New Figure 4E, F, G). This is consistent with uptake of HLA-G by the C28-C74 form of KIR2DL4 and with a switch from C10-C28 to C28-C74 catalyzed by PDI.

Furthermore, inhibition of PDI caused a decrease of C28-C74 KIR2DL4 at the cell surface of primary NK cells (New Figure 5E, F). This data is consistent with a requirement for a switch from C10-C28 to C28-C74 catalyzed by PDI, which maintains a pool of C28-C74 KIR2DL4 at the cell surface for HLA-G binding and internalization. As shown in the original Figure 3, PDI can reduce the C10-C28 bond in purified WT KIR2DL4 in vitro.

**Recommendations for the authors:**

**Reviewing Editor Comments:**

To improve the strength of the evidence and the overall impact of the paper, please address the following major points:

(1) Validation in Primary Cells:

The central biological framing of the paper involves decidual NK cell responses to soluble HLA-G. We strongly recommend performing a critical experiment using primary NK cells to test whether PDI inhibition or thiol blockade alters KIR2DL4 surface retention and HLA-G uptake in a manner consistent with your observations in 293T cells.

We have added new experiments with primary, resting NK cells, as described in our response to the major point 3 of reviewer #1, and to the weakness raised by reviewer #2.

Briefly, we have included experiments in the revised manuscript on the effect of PDI inhibition on HLA-G uptake in primary NK cells (New Figure 4E, F, G) and on transient accumulation of KIR2DL4 at the cell surface (in a C28-C74 bonded form) of primary NK cells (New Figure 5E, F). The data showed that HLA-G endocytosis by primary NK cells and the presence of KIR2DL4 at the plasma membrane of primary NK cells were reduced after inhibition of PDI.

(2) Clarification of the "Switching" Mechanism:

The current data points toward the coexistence of the Cys10-Cys28 and Cys28-Cys74 states. Please clarify or provide evidence regarding whether a dynamic conversion occurs (e.g., prior to binding, upon ligand engagement, or during trafficking) versus a model of stable coexistence of two distinct receptor pools.

Stable coexistence of two distinct KIR2DL4 receptor pools was a plausible hypothesis but one that is not supported by some of our data. In such a scenario, the C10-C28 form would not bind HLA-G and would reside in endosomes. It could have a role that is not related to HLA-G nor to the transcriptional response induced by HLA-G. However, our recent paper [2] showed that the transcriptional response of primary NK cells to soluble mAb #33 (bound to C10-C28) is very similar (R^2^=0.89) to that of resting NK cells incubated with soluble HLA-G (bound to C28-C74). These two ligands were tested at the same time, at the same molarity, and with the same primary NK cells [2].

We don’t have answers yet to some obvious questions: is there switching after internalization of KIR2DL4 bound to mAb #33? What is the fate of C28-C74 that internalizes with HLA-G? We are not aware of technology that would answer these questions.

A C28-C74 form, as a separate pool with residency at the cell surface, could be functional and respond to HLA-G by internalization and signaling from endosomes. However, there is no stable pool of C28-C74 KIR2DL4 at the cell surface and C28-C74 is depleted from the cell surface in the presence of PDI inhibitor (new Figure 5E, F), suggesting that C28-C74 KIR2DL4 is generated by the activity of PDI (new Figure S5). The sum of our experiments points to a tightly regulated control of KIR2DL4 biology, rather than the coexistence of two separate pools. A separate pool of C10-C28 KIR2DL4 would remain in an inactive state as far as the response to HLA-G is concerned. We favor the model whereby functional C28-C74 is generated from C10-C28 by the activity of PDI.

Why could the response to HLA-G not be simpler? We address this point in the Discussion. One reason is that C28-C74 KIR2DL4 signaling at the plasma membrane of NK cells could be subject to inhibition by LILRB1 and NKG2A-CD94, co-expressed on NK cells, which bind to HLA-G and HLA-E, respectively, on fetal trophoblasts that encounter maternal NK cells in the decidua. These inhibitory receptors are known to be dominant against activation signals [3]. Trophoblast cells that invade the maternal decidua and encounter decidual NK cells selectively express HLA-C, HLA-E, and HLA-G. Strong inhibition signals by LILRB1 and NKG2A-CD94 could prevent activation through KIR2DL4. However, KIR2DL4 signaling, which occurs in endosomes [4] where signaling is sustained [5], can bypass these inhibitory signals at the plasma membrane.

(3) Specificity of the PDI Model:

Please elaborate on the relevance of extracellular PDI. Specifically, how does PDI perturbation affect the relative abundance of the two disulfide forms in a cellular context?

We show in Figure 5E that two mAb for KIR2DL4 recognize different forms of the receptor. While mAb #33 recognizes only the C10-C28 form of the receptor, which is not at the cell surface, mAb 2238 recognizes both forms of the receptor. This allowed us to examine the effect of PDI on surface expression of the C28-C74 form of KIR2DL4 as detected by mAb 2238. We show that PDI inhibition reduces surface staining of C28-C74 (new Figure 5F), consistent with a model whereby a switch from C10-C28 to C28-C74 is catalyzed by PDI.

A quantitative assessment of the relative abundance of the two forms of KIR2DL4 upon inhibition by PDI in a cellular context would have to be carried out by mass spec analysis of the two forms before and after treatment. That would be a very challenging experiment to perform with intact cells rather than purified proteins.

(4) Agonist Antibody Mechanism:

The manuscript mentions mAb #33 as a KIR2DL4 agonist. It would be highly informative for the reader if you could elaborate on whether this antibody activates the receptor by stabilizing a specific disulfide state or by driving internalization independently of HLA-G.

We have shown that the agonist mAb #33 recognizes only the C10-C28 form (Figure 5E). We do not yet understand how it activates KIR2DL4. We do know that mAb #33 is not driving internalization considering that the receptor internalizes constitutively and is predominantly located in endosomes in the absence of HLA-G. Instead, it is the C10-C28 form of the receptor that carries mAb #33 into endosomes. Understanding how mAb #33 may function as a receptor agonist will require crystallization of the antibody bound to the receptor and is beyond the scope of this study. Structural studies of KIR2DL4 have been very difficult, due in part to its isoforms and tendency to form oligomers. It is not possible to answer your interesting question at this time.

Minor Revisions:

(1) Imaging Quantification:

Ensure all figure legends include the number of independent experiments (n), specific statistical tests used, and precise alignment with the Methods section.

This information is now included in the Methods section.

(2) Textual Flow:

To enhance engagement, please integrate the logic of Table 1 more explicitly into the main text of the Results section.

This has been done.

(3) Structural Discussion:

Acknowledge the limitations of using structure prediction for the binding interface and discuss how these models align with existing literature on KIR-ligand interactions.

We have described the use of AlphaFold solely as a tool to make predictions. Predictions can be wrong. Even so, they can generate new and useful hypotheses, as they did here. Existing, traditional KIR-ligand interactions are not informative in the context of the D0 domain in KIR2DL4 for the following reasons:

The KIR2DL1/2/3 receptors with 2 Ig domains (hence 2D) have a D1 and a D2 domain. A comparison with KIR2DL4, which has a D0 and a D2 domain, may not be informative.

The KIR3D receptors have the three domains, D0, D1 and D2. A structure of KIR3DL1 bound to HLA-B has been solved [6] by our collaborator for the revision, Dr. Jamie Rossjohn. As shown and mentioned in our manuscript (Fig. S7C and Legend), “predicted” contacts of the KIR2DL4 D2 domain with HLA-G involve residues conserved in the heavy chains of HLA-B and HLA-G and residues conserved in the KIR3DL1 and KIR2DL4 D2 domains. It is therefore likely that the KIR2DL4 D2 domain contacts HLA-G in a similar way.

As for the KIR2DL4 D0 domain, it is very different. Due to the similarity between D2 domains of KIR3DL1 and KIR2DL4, and to the lack of a D1 domain in KIR2DL4, the KIR2DL4 D0 domain is in a completely different space than the D0 domain of KIR3DL1. “Predictions” by AlphaFold show that there could be interactions between the KIR2DL4 D0 domain and HLA-G (Figures 7 and S7). These predictions could be wrong. Nevertheless, the disulfide switch in the KIR2DL4 D0 domain correlates with a predicted change elsewhere on D0 at a position compatible with proximity to HLA-G. Furthermore, the KIR2DL4 isoform with a Cys28-Cys74 bond is “predicted” to be more aligned with a potential binding site than the Cys10-Cys28 isoform. Having no structural guide as a reference on how KIR2DL4 D0 domain may interact with HLA-G, such predictions may generate testable hypotheses.

As we clearly state in the manuscript: “Structures of KIR2DL4–HLA-G complexes obtained experimentally are required to determine how HLA-G distinguishes the D0 domain in the alternative disulfide-bonded configurations.” (Results), and “Rules that dictate HLA-G binding to KIR2DL4 await further studies and structures of KIR2DL4–HLA-G complexes.” (Discussion).

In the revised manuscript, we have now included SPR binding data for KIR2DL4 with HLA-G. We also show a higher affinity of HLA-G for the C28-C74 form of KIR2DL4. This has strengthened the study as it validates our model whereby switching to the functional form of the receptor allows binding of HLA-G. In this regard, we also include data showing that only the C28-C74 form of KIR2DL4 can respond to HLA-G to induce transcription of an ISG response. This provides a functional correlate to the role of the different disulfide forms of the receptor.

**Reviewer #2 (Recommendations for the authors):**

Major points to address:

(1) Exhibit relevance using primary NK cells (using primary NK). The central biological framing is decidual NK responses to soluble HLA-G during early pregnancy, yet most mechanistic work is in 293T transfectants. The authors can perform one of the critical experiments using primary NK cells with soluble HLA-G stimulation. They should test whether PDI inhibition/thiol blockade similarly alters KIR2DL4 surface retention and HLA-G uptake in primary NK cells

These experiments have been performed in primary NK cells and are described in the new Figure 4E, F, G and Figure 5F.

(2) The authors should detail more about the relevance of extracellular PDI and the effect of PDI perturbation on the abundance of the two disulfide forms in cells. They should also provide evidence or discuss whether switching occurs prior to ligand binding, upon ligand engagement, or during trafficking.

Such experiments would be very challenging. The predicted structural change is minor and may not be detectable by changes in proximity of labeled reporters. Ligand is not required for switching. We do know that PDI can convert C10-C28 into C28-C74, presumably by accessibility to the KIR2DL4 Cys28 when bonded in a C10-C28 configuration (Figure 2). How ligands (mAb #33 or HLA-G) impact KIR2DL4 structure is unknown. Data are compatible with the possibility of a stabilization of C10-C28 by mAb #33 and of C28-C74 by HLA-G.

(3) The authors should elaborate on whether mAb #33 activates by stabilizing or by driving internalization independent of HLA-G. This is very interesting to the reader, given mAb #33 as a KIR2DL4 agonist.

The question is undeniably interesting. mAb #33 is not required for internalization but is required for signaling. The C10-C28 KIR2DL4 configuration to which it binds internalizes constitutively and resides mainly in endosomes. How mAb #33 internalization by KIR2DL4 (not the reverse) results in signaling is not known. Nor is it known for the alternative form, C28-C74, which binds HLA-G, internalizes it, and signals for a transcriptional response very similar to that of C10-C28 bound to mAb #33 [2]. The C28-C74 KIR2DL4 configuration is retained, probably transiently, at the cell surface, to be available for HLA-G binding and internalization.

Minor points to address:

(1) The authors should ensure that all imaging quantifications include n, the number of experiments, and statistical treatment. Some are described in the Methods section. Please align figure legends with the method in detail.

Details of the imaging experiments are provided in the Methods section.

(2) Please summarize the Table 1 logic in the main text for enhanced reader engagement.

This has been done.

(3) The authors identify both Cys10-Cys28 and Cys28-Cys74 states in human cells. The data points towards coexistence rather than towards dynamic conversion. Please provide clarity on switching versus stable coexistence of two forms.

Stable coexistence of two distinct KIR2DL4 receptor pools was a plausible hypothesis but one that is not supported by some of our data. In such a scenario, the C10-C28 form would not bind HLA-G and would reside in endosomes. It could have a role that is not related to HLA-G nor to the transcriptional response induced by HLA-G. However, our recent paper [2] showed that the transcriptional response of primary NK cells to soluble mAb #33 (bound to C10-C28) is very similar (R^2^=0.89) to that of resting NK cells incubated with soluble HLA-G (bound to C28-C74). These two ligands were tested at the same time, at the same molarity, and with the same primary NK cells [2].

We don’t have answers yet to some obvious questions: is there switching after internalization of KIR2DL4 bound to mAb #33? What is the fate of C28-C74 that internalizes with HLA-G? We are not aware of technology that would answer these questions.

A C28-C74 form, as a separate pool with residency at the cell surface, could be functional and respond to HLA-G by internalization and signaling from endosomes. However, there is no stable pool of C28-C74 KIR2DL4 at the cell surface and C28-C74 is depleted from the cell surface in the presence of PDI inhibitor (new Figure 5E, F), suggesting that C28-C74 KIR2DL4 is generated by the activity of PDI (new Figure S5). The sum of our experiments points to a tightly regulated control of KIR2DL4 biology, rather than the coexistence of two separate pools. A separate pool of C10-C28 KIR2DL4 would remain in an inactive state as far as the response to HLA-G is concerned. We favor the model whereby functional C28-C74 is generated from C10-C28 by the activity of PDI.

Why could the response to HLA-G not be simpler? We address this point in the Discussion. One reason is that C28-C74 KIR2DL4 signaling at the plasma membrane of NK cells could be subject to inhibition by LILRB1 and NKG2A-CD94, co-expressed on NK cells, which bind to HLA-G and HLA-E, respectively. These inhibitory receptors are known to be dominant against activation signals [3]. Trophoblast cells that invade the maternal decidua express HLA-E and HLA-G and encounter decidual NK cells that express LILRB1 and NKG2A-CD94. Strong inhibition signals induced by these two receptors could prevent activation through KIR2DL4. KIR2DL4 signaling in endosomes protects it from these inhibitory signals and benefits from the sustained signaling property of endosomal signaling platforms [5].

(1) S. Moradi et al., The structure of the atypical killer cell immunoglobulin-like receptor, KIR2DL4. J Biol Chem 290, 10460-10471 (2015).

(2) S. Rajagopalan et al., The fetal trophoblast cell marker HLA-G activates a type I interferon response in primary NK cells through the receptor KIR2DL4. Sci Signal 19, eadv2400 (2026).

(3) E. O. Long, H. S. Kim, D. Liu, M. E. Peterson, S. Rajagopalan, Controlling natural killer cell responses: integration of signals for activation and inhibition. Annu Rev Immunol 31, 227-258 (2013).

(4) S. Rajagopalan et al., Activation of NK cells by an endocytosed receptor for soluble HLA-G. PLoS Biol 4, e9 (2006).

(5) M. Miaczynska, L. Pelkmans, M. Zerial, Not just a sink: endosomes in control of signal transduction. Curr Opin Cell Biol 16, 400-406 (2004).

(6) J. P. Vivian et al., Killer cell immunoglobulin-like receptor 3DL1-mediated recognition of human leukocyte antigen B. Nature 479, 401-405 (2011).
